# Combined fetal fraction to analyze the Z-score accuracy of noninvasive prenatal testing for fetal trisomies 13, 18, and 21

**DOI:** 10.1007/s10815-022-02694-8

**Published:** 2023-02-10

**Authors:** Jiexia Yang, Jing Wu, Dongmei Wang, Yaping Hou, Fangfang Guo, Qi Zhang, Haishan Peng, Yixia Wang, Aihua Yin

**Affiliations:** 1 Medical Genetic Centre, Guangdong Women and Children Hospital, Guangzhou, 511400 Guangdong China; 2 Maternal and Children Metabolic-Genetic Key Laboratory, Guangdong Women and Children Hospital, Guangzhou, 511400 Guangdong China; 3 Department of Prenatal Diagnosis Center, Guangdong Women and Children Hospital, No. 521 Xingnan Road, Panyu District, Guangzhou, 511400 China

**Keywords:** Noninvasive prenatal testing, Z-score, Fetal fraction, Positive predictive value (PPV), Post-test consultation

## Abstract

**Objective:**

This study aims to evaluate the correlation combined fetal fraction and Z-score for fetal trisomies 13, 18, and 21 of NIPT by the semiconductor sequencing platform and further analyze the differences of different sequencing depths.

**Methods:**

A cohort of 61,581 pregnancies were recruited for NIPT. Invasive prenatal diagnostic confirmation is recommended in all high-risk NIPT cases. Logistic regression and rank correlation analysis were applied to analyze the relationship between different parameters. ROC curve analysis was adopted to analyze the cutoff values of Z-score and fetal fraction.

**Results:**

A total of 278 common trisomy pregnancies were verified in 377 NIPT-positive results. The fitted logistic regression models revealed that Z-scores of NIPT-positive results were significantly associated with PPVs (*p* < 0.05). The ROC curve analysis showed that the optimal cutoff value of Z-scores for T21, T18, and T13 was 7.597, 4.944, and 9.135 for NIPT and 9.489, 8.004, and 12.4 for NIPT-plus. If combing fetal fraction as another evaluation factor, the PPV of trisomy 21 gradually improved. We analyzed the correlation between the fetal fraction and the PPV, which revealed that the fetal fraction was significantly correlated with PPV. By analyzing the PPV of different groups divided by the associated criteria obtained from ROC curve, the PPV of high Z-score and high fetal fraction is higher in groups of Z-score > the optimal cutoff value.

**Conclusion:**

The results of this study show that the fetal fraction is significantly correlated with the PPV. Combining fetal fraction with Z-score is significantly better than in groups of Z-score-associated criteria; clinicians can give more accurate and efficient prenatal genetic counseling.

Since 1997, the discovery of cell-free fetal DNA (cffDNA) has opened a new avenue for noninvasive prenatal testing (NIPT) [1]. Due to the wide application of the next-generation sequencing (NGS), NIPT has been widely used in the screening of fetal trisomies of 21, 18, and 13 [2–5]. Although NIPT has been proven to be currently the best noninvasive method for the diagnosis of common fetal aneuploidy, it remains a screening tool because cffDNA is mainly from the placenta rather than the fetus. There may be a discrepancy in genetic material between the placenta and the fetus [6, 7]. Therefore, further confirmation of invasive prenatal diagnosis is necessary.

Z-score represents the risk value of NIPT; Z-score (also called standard score) describes the position of a raw score in terms of its distance from the mean. The Z-score refers to the number of standard deviations from the mean of a reference data set [8]. A significant correlation between Z-score and positive predictive value (PPV) has been reported in many literatures [9], [10]. Some literatures also suggests that fetal fraction is crucial to the accuracy of the results [11, 12]. Indicating that accurate assessment of cffDNA concentration is an important prerequisite.

In this study, semiconductor sequencing platform with average read length of over 100 bp was used to sequence the complete cell-free DNA fragments in the peripheral blood [13]. In addition, fetal fraction can be calculated without being restricted by fetal gender [14]. To study the internal relationship between fetal fraction and PPV. Logistic regression and receiver operating characteristic (ROC) curve were adopted to evaluate the Z-score accuracy of NIPT for trisomies 21, 18, and 13 and to analyze the relationship between Z-score and PPV performance. The associations between Z-score and other parameters were analyzed to discover relevant factors.

## Materials and methods

### Participant recruitment

This study was approved by the Ethics Review Committee of Guangdong Women and Children Hospital (number 2013102301). All clinical work procedures are carried out in accordance with the relevant guidelines and practices. In consultation with physicians, subjects were informed of the objectivity, accuracy, consequence, and limitation of NIPT, and each participant signed an informed consent.

A total of 61,581 pregnant women who undergone NIPT in Guangdong Woman and Children Hospital from January 2015 to December 2020 were recruited for the retrospective study. After detailed genetic counseling by the doctor, according to their own conditions, pregnant women voluntarily choose 0.15 × NIPT or 0.4 × NIPT (NIPT-plus).

### Sample preparation, DNA sequencing, and bioinformatics analysis

For each subject, a peripheral blood sample (5 ml) was withdrawn from the cubital vein using an EDTA K_2_ tube. The blood was centrifuged at 4 °C and 1,600 × g for 10 min by Eppendorf 5810R (Eppendorf, Germany). Then the plasma was transferred to another tube and centrifuged at 4 °C and 16,000 × g for 10 min by Eppendorf 5424 centrifuge (Eppendorf, Germany). The supernatant were stored at − 20 °C as soon as possible until genomic DNA is extracted.

DNA enrichment was performed after end-repairing with an average particle size of 1 um for the purpose of selecting fragments smaller than 160 bp [14]. The tubes were vibrated for 3 s after adding the beads. After 5 min, transfer the tubes to the magnetic rack. The supernatant was used for adaptor ligation.

The next-generation sequencing was performed using semiconductor sequencing technique on the Bioelectronseq 4000 sequencing platform (CFDA registration permit NO. 20153400309, CapitalBio, China). As detailed in our previous article [13], a total of 9–23 libraries were pooled and sequenced in the range of ~ 200 bp reads. This study included two different sequencing depth methods. The sequencing depths of NIPT and are about 0.15 × and 0.4 × , and the data volumes are 3 million and 8 million of reads, respectively.

Combined GC correction and Z-score testing methods were used to identify fetal chromosome aneuploidy of trisomies 21, 18, and 13 as described previously [13]. The fetal and maternal chromosome aneuploidy was classified using modified Stouffer’s Z-score method. The Z‐score obeyed standard normal distribution; Z-scores were used to evaluate the risk of chromosomal aneuploidies which was set within the range from − 3 to 3. If Z‐score > 3 or Z‐score <  − 3, the sample was classified as a high risk of chromosomal aneuploidies. On the contrary, if the Z‐score was between − 3 and 3, the sample was divided to have a low risk.

To estimate the ratio of cffDNA in maternal peripheral blood, two types of methods were used [14]. Briefly, read proportion of the Y chromosome was used for a male fetus, and the LOESS regression was applied for a female fetus.

### Prenatal diagnosis and pregnancy follow-up

Professional genetic counseling was given for pregnant women of high risk in NIPT. After fully informed, prenatal diagnosis to obtain fetal cells through puncture (villus, amniotic fluid, or umbilical cord blood) for fetal chromosome analysis was conducted. Patients at high risk for NIPT were confirmed by karyotyping (G-band resolution 400 bands) or chromosome microarray analysis (CMA) (CytoScanTM 750 K, Affymetrix, USA). All participants were followed up through telephone interviews for neonatal outcomes and growths.

### Statistics

SPPS 22.0 were used for data statistical analysis. Logistic regression was used to analyze the relationship between the Z-score and the PPV performance. Python language was applied to carry on logistic regression analyses. The differences between rates were tested by Chi‐square test or Fisher exact tests. Results with *P* values < 0.05 were considered statistically significant. The associated criteria, specificity, and sensitivity were calculated by ROC curve analyses using MedCalc (Wan et al., 2021; Zhou et al., 2021). Correlation between Z-score and maternal age, gestational age, fetal fraction, or BMI was conducted by rank analyses using SPSS.

## Results

### Population profiles and NIPT results

Among the 61,581 cases who underwent NIPT, 49,393 adopted 0.15 × sequencing (NIPT), and 12,188 adopted 0.4 × sequencing (NIPT-plus). The basic characteristics and general results were listed in Table 1. A total of 377 were found to be trisomy positive and underwent invasive prenatal diagnosis of chromosomal microarray analysis (CMA) or chromosome karyotyping (CS). Among them, 278 were confirmed to have fetuses with trisomy, in which 213 were T21, 48 were T18, and 17 were T13. Under set conditions, the overall PPV was 73.74%. The PPV of T21, T18, and T13 were 84.80%, 69.23%, and 25.00% for NIPT, and the PPV of T21, T18, and T13 were 86.96%, 80.00%, and 35.00% for NIPT-plus.

**Table 1:** Demographic characteristics of 377 positive cases examined by NIPT or NIPT-plus

Characteristic		NIPT/NIPT-plus positive cases	True positive	False positive	Maternal age (year)	Gestational age (week)	Fetal fraction(%)	BMI
NIPT	Trisomy 21	204	173	31	30.05 ± 5.25	18.76 ± 4.93	14.96 ± 6.47	22.83 ± 2.98
	Trisomy 18	52	36	16	31.85 ± 5.44	16.81 ± 4.03	12.23 ± 4.64	22.52 ± 2.83
	Trisomy 13	40	10	30	32.85 ± 5.96	16.99 ± 3.68	15.57 ± 6.31	22.43 ± 3.16
NIPT-plus	Trisomy 21	46	40	6	31.59 ± 6.35	16.23 ± 3.82	17.94 ± 6.94	22.41 ± 3.19
	Trisomy 18	15	12	3	32.46 ± 5.80	18.00 ± 3.19	13.89 ± 2.70	23.62 ± 3.36
	Trisomy 13	20	7	13	31.53 ± 5.24	17.22 ± 4.73	19.75 ± 8.24	22.53 ± 3.67

Abbreviations: NIPT, noninvasive prenatal testing; BMI, body mass index

### Analysis of logistics regression and ROC curve

**Fig. 1:**
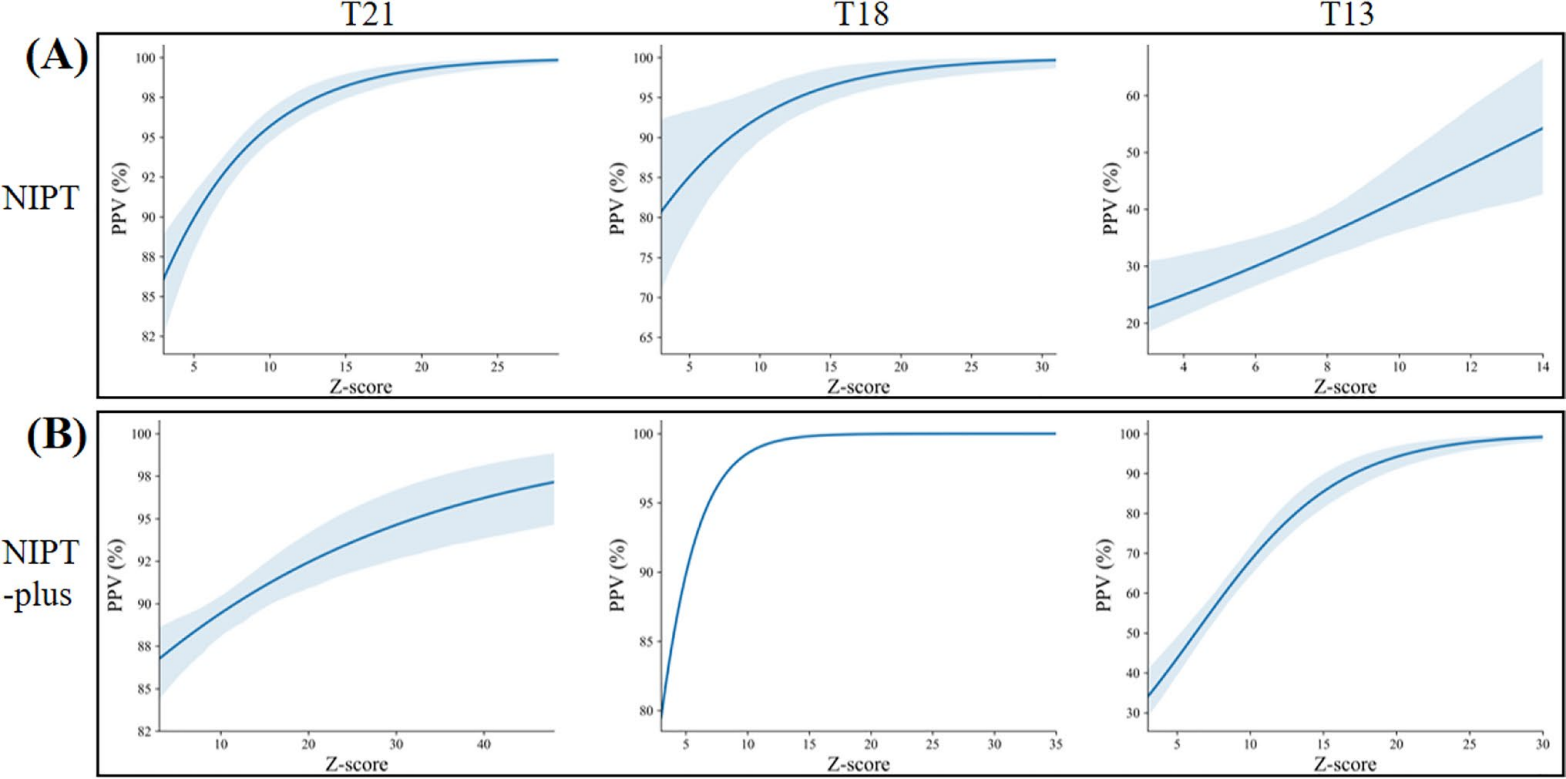
The fitted logistic regression analysis of Z-scores and PPVs, The fitted logistic regression models for the PPV with 95% CIs, the fitted logistic regression models revealed that Z-scores of NIPT/NIPT-plus positive results were positively related to true positive results (P ＜0.05).

**Fig. 2:**
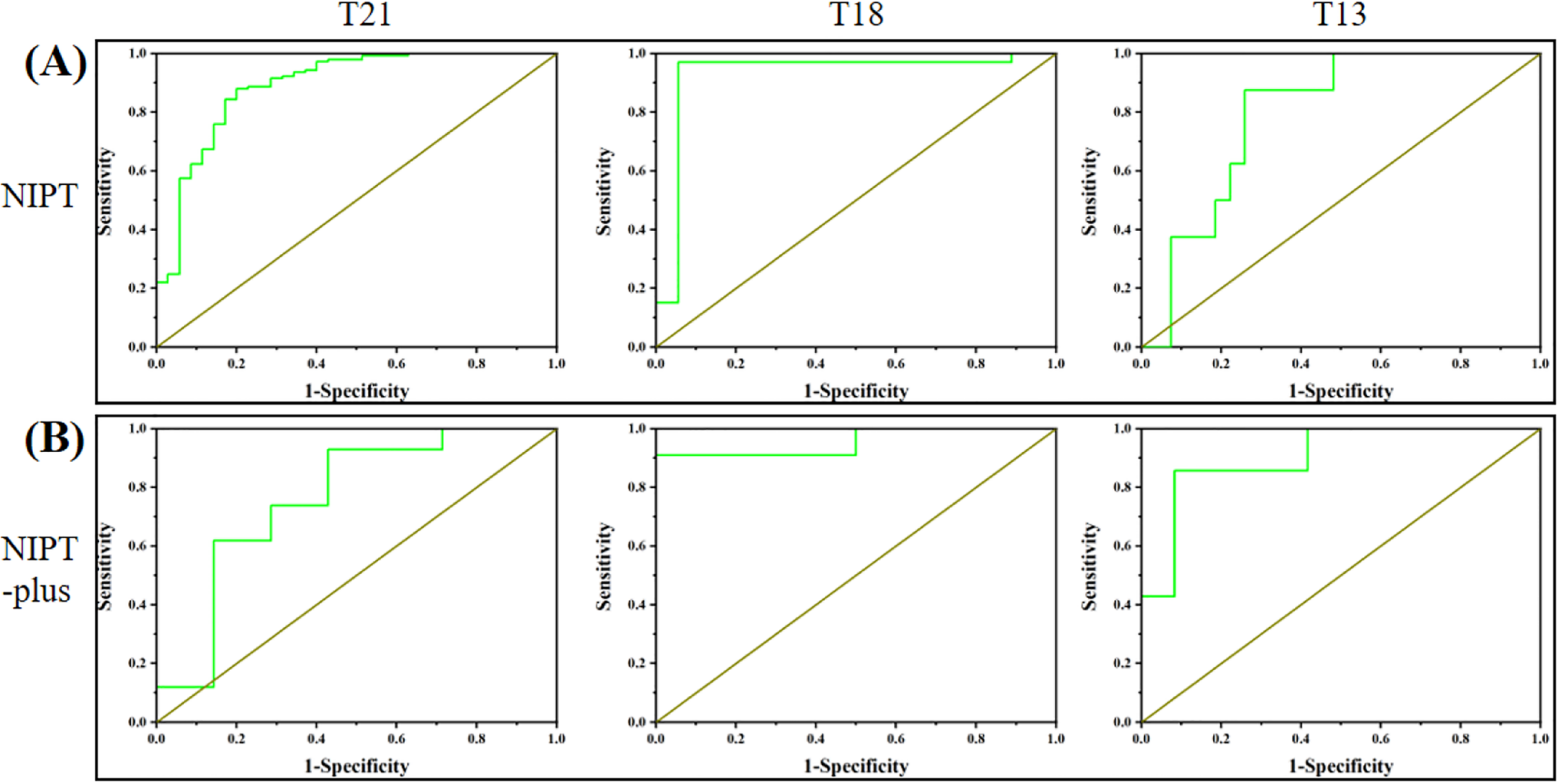
The ROC curve analysis of T21, T18, and T13. For NIPT, the areas under the ROC curves were 0.891, 0.928, and 0.796, The results showed that the sensitivity and specificity for T21 were 87.94% and 80%, for T18 were 96.94% and 94.44%, and for T13 were 87.50% and 74.07%. For NIPT-plus, the areas under the ROC curves were 0.762, 0.955, and 0.905, The results showed that the sensitivity and specificity of T21 were 92.86% and 57.14%, the sensitivity and specificity of T18 were 90.91% and 100%, and the sensitivity and specificity of T13 were 85.71% and 91.67%. NIPT, noninvasive prenatal testing.

As shown in Fig. 1, the fitted logistic regression models revealed that Z-scores of NIPT/NIPT-plus positive results were positively related to true positive results (*P* < 0.05). For NIPT, ROC curve analysis showed that the optimal cutoff value of Z-scores for predication of T21, T18, and T13 was 7.597, 4.944, and 9.135; the areas under the ROC curves were 0.891, 0.928, and 0.796; and the Youden index J was 0.6794, 0.9141, and 0.6157, respectively (Fig. 2 and Table 2). The results showed that the sensitivity and specificity for T21 were 87.94% and 80%, for T18 were 96.94% and 94.44%, and for T13 were 87.50% and 74.07%. For NIPT-plus, ROC curve analysis showed that the optimal truncation value of Z-scores for predication of T21, T18, and T13 was 9.489, 8.004, and 12.4; the areas under the ROC curves were 0.762, 0.955, and 0.905; and the Youden index J was 0.5000, 0.9091, and 0.7738, respectively (Fig. 2 and Table 2). The results showed that the sensitivity and specificity of T21 were 92.86% and 57.14%, the sensitivity and specificity of T18 were 90.91% and 100%, and the sensitivity and specificity of T13 were 85.71% and 91.67%.

**Table 2:** Results of ROC curve analysis of positive results in NIPT and NIPT-plus

Characteristic	NIPT			NIPT-plus		
	Trisomy 21	Trisomy 18	Trisomy 13	Trisomy 21	Trisomy 18	Trisomy 13
Area under the ROC curve (AUC)	0.8910	0.9280	0.7960	0.7620	0.9550	0.9050
Standard error	0.0350	0.0525	0.0775	0.1230	0.0643	0.0734
95% confidence interval	0.836–0.933	0.819–0.981	0.626–0.913	0.619–0.872	0.683–1.000	0.682–0.990
Significance level *P* (area = 0.5)	< 0.0001	< 0.0001	0.0001	0.0337	< 0.0001	< 0.0001
Youden index J	0.6794	0.9141	0.6157	0.5000	0.9091	0.7738
Optimal Z-score cutoff value	7.597	4.944	9.135	9.489	8.004	12.4
Sensitivity	87.94%	96.97%	87.50%	92.86%	90.91%	85.71%
Specificity	80%	94.44%	74.07%	57.14%	100%	91.67%

Abbreviations: AUC, area under curve; NIPT, noninvasive prenatal testing; ROC, receiver operating characteristic curve

### Correlation analysis between Z-score and relative parameters

As Z-score was significantly correlated with PPV, the correlation between Z-score and other parameters were further studied. Rank correlation analysis between Z-score and maternal age, gestational age, fetal fraction, or BMI was conducted (Table 3). The spearman’s coefficient of rank correlation of between Z-score and fetal fraction in T21 of NIPT and NIPT-plus was 0.60 (*P* < 0.0001, 95% CI 0.49–0.69) and 0.82 (*P* < 0.0001, 95% CI 0.70–0.90). The Spearman’s coefficient of rank correlation between Z-score and BMI in T21 of NIPT was − 0.28 (*P* = 0.0001, 95% CI − 0.414– − 0.141). These results indicate that the fetal fraction was significantly correlated with Z-score in T21 of NIPT and NIPT-plus. A weak correlation was found between Z-score and BMI in T21 of NIPT. No significant correlation was found in T18 and T13 of NIPT and NIPT-plus, which may be due to the small sample size. It is worth mentioning that no significant correlation was found between fetal fraction and PPV.

**Table 3:** The correlation between Z-score and relative parameters

Characteristic		Relevant parameters for correlation analysis with Z-score	Spearman’s coefficient of rank correlation (rho)	Significance level (*P* value)^*^	95% confidence interval for rho
NIPT	T21	Maternal age (year)	− 0.1300	0.0862	− 0.273–0.0186
		Gestational age (week)	− 0.0236	0.7563	− 0.171–0.125
		Fetal fraction	0.5970	< 0.0001	0.493–0.685
		BMI	− 0.2830	0.0001	− 0.414– − 0.141
	T18	Maternal age (year)	− 0.2530	0.0797	− 0.499–0.0306
		Gestational age (week)	0.1360	0.3416	− 0.145– 0.397
		Fetal fraction	0.0520	0.7173	− 0.227–0.323
		BMI	− 0.1660	0.2433	− 0.423–0.114
	T13	Maternal age (year)	0.0380	0.8282	− 0.299–0.367
		Gestational age (week)	− 0.00998	0.9546	− 0.342–0.324
		Fetal fraction	0.0656	0.7083	− 0.274–0.390
		BMI	0.1620	0.3523	− 0.181–0.470
NIPT-plus	T21	Maternal age (year)	− 0.0300	0.8380	− 0.309–0.253
		Gestational age (week)	0.2120	0.1434	− 0.0734–0.466
		Fetal fraction	0.8220	< 0.0001	0.703–0.896
		BMI	− 0.1310	0.3696	− 0.398–0.156
	T18	Maternal age (year)	0.2290	0.4524	− 0.369–0.692
		Gestational age (week)	− 0.1440	0.6392	− 0.644–0.442
		Fetal fraction	0.2480	0.4147	− 0.351–0.703
		BMI	− 0.2990	0.3204	− 0.730–0.301
	T13	Maternal age (year)	0.0503	0.8380	− 0.413–0.493
		Gestational age (week)	− 0.1970	0.4200	− 0.597–0.283
		Fetal fraction	0.2140	0.3783	− 0.266–0.609
		BMI	− 0.2090	0.3905	− 0.606–0.271

^*^the *P* value < 0.0001 indicates a significant correlation

Abbreviations: NIPT, noninvasive prenatal testing; BMI, body mass index

Based on the above results, fetal fraction was taken into consideration when calculating the PPV based on Z-score. Due to the few data of T18 and T13, only T21 of NIPT and NIPT-plus were analyzed. The cases were first divided into two groups based on the associated criterion of Z-scores: the low Z-score (LZ) (Z-score < associated criterion) and high Z-score (HZ) groups (Z-score ≥ associated criterion). Then, the cases were divided into two groups based on associated criterion of ROC curves: the low criterion (LC (fetal fraction < associated criterion) and high criterion (HC) groups (fetal fraction ≥ associated criterion). The PPV of each group was calculated (Table 4). Nonparametric test of Mann–Whitney was used to test the difference significance between LC and HC, and *Z* and *P* values were shown. Compared with LC groups, HC groups have a higher PPV in HZ groups of NIPT and NIPT-plus. But no significant difference was found between LC groups and HC groups. No similar results were found between the HC and LC groups in LZ groups of NIPT and NIPT-plus.

**Table 4:** Comparison of the positive predictive values of NIPT and NIPT-plus positive results with T21

Characteristic	NIPT				NIPT-plus			
	LZ		HZ		LZ		HZ	
	LC	HC	LC	HC	LC	HC	LC	HC
PPV (%)	42.86 (9/21)	34.78 (8/23)	89.29 (25/28)	95.19 (99/104)	75.00 (3/4)	0.00 (0/3)	75.00 (3/4)	94.74 (36/38)
*Z*	− 0.54		− 1.158		− 1.837		− 1.440	
Significance level (*P* value) ^*^	0.587		0.247		0.066		0.150	

the associated criterion of *Z*-score of T21 were 7.597 for NIPT and 9.489 for NIPT-plus as shown in Table 2. The associated criteria of fetal fraction of T21 were 12.1 for NIPT and 13.9 for NIPT-plus

^*^the *P* value < 0.05 indicates a significant difference

Abbreviations: NIPT, noninvasive prenatal testing; LZ, low Z-score; HZ, high Z-score; LC, low criterion; HC, high criterion

## Discussion

NIPT has been widely used in prenatal screening with excellent specificity and sensitivity [15]. PPV is often used to evaluate the accuracy of NIPT performance [16]. However, detailed genetic counseling before and after testing is much more important. Pre-test consultation can be carried out according to relevant international and domestic guidelines [17]. But it is difficult for doctors to give appropriate judgment for every high-risk outcome in post-test consultation.

In this study, both NIPT and NIPT-plus showed excellent sensitivity and specificity. Logistic regression and ROC analysis showed that the PPV of NIPT and NIPT-plus was significantly correlated with Z-score, which is consistent with other reports [9, 10]. Therefore, clinicians can first give effective and objective consultation opinions according to Z-score for high-risk patients.

Further, by analyzing the correlation between Z-score and other parameters, fetal fraction was found to be significantly correlated with the PPV in T21. Dividing the test results into two groups according to cutoff values of fetal fraction and Z-score showed a clear distinction of positive predictive value. No significant difference was found, which might due to insufficient statistical power. The fetal fraction is directly related to PPV, suggesting that higher fetal fraction enables to obtain more valid data and further more accurate results. Therefore, fetal fraction can be used as another reference following Z-Score for doctors’ post-test consultation. In addition, despite numerous reported that the fetal fraction increases with gestational age [18], no correlation between gestational age and Z-score was found in this study.

As cffDNA in maternal plasma was shorter than maternal cfDNA, some literatures reported that sequence shorter cfDNA fragments decreases the false negative rate of noninvasive prenatal testing [19, 20]. Therefore, the proportion of cell‐free fetal DNA (cffDNA) in extracted DNA is very important for NIPT result. In this study, cffDNA enrichment technology was used to increase the average cffDNA concentration from 10 to 20% and significantly reduce false negatives [14]. When the Z-score is between 3 and 5, the PPV of T21 high-risk is less than 20%. In theory, the chance of overturning is greater than 80%. In addition, it is also very important to evaluate the risk by combing with the fetal fraction. For example, the fetal fraction is 30%, but the *Z* value is only more than 4 points. The possibility of placental mosaicism is more higher [21], and the probability that the fetus itself is normal is relatively high. False positives and false negatives are unavoidable problems for NIPT [22]. Fetal cell-free DNA comes from the placenta, which develops from embryonic trophoblast cells. Due to the different cell sources of the placenta and fetal DNA, there is inconsistency in DNA materials. This phenomenon is called confined placental mosaicism (CPM), and the incidence of CPM can reach 1–3% [23].

Although many studies reported that the PPV of NIPT was significantly correlated with Z-score, few studies have involved fetal fraction. This study proposes that the fetal fraction is significantly correlated with the PPV, especially trisomy 21. The effect of fetal fraction was more pronounced in the low Z-score group. Combined with fetal fraction and Z-score analysis, clinicians can give more pertinent recommendations. The shortcoming of this study is that the sample size of the low Z-score group is small. The next plan is to accumulate more data for analysis and further confirm the current results.

In a word, there is a significant correlation between fetal fraction and PPV of NIPT. Combining fetal fraction with Z-score is significantly better than in groups of Z-score-associated criteria; clinicians can give more accurate and efficient prenatal genetic counseling.

## Data Availability

The datasets used and/or analyzed during the current study are available from the corresponding author on reasonable request.

## Abbreviations

*NIPT *: Noninvasive prenatal testing
*PPV *: Positive predictive value
*cffDNA *: Cell-free fetal DNA
*ACMG *: American Society of Medical Genetics and Genomics
*CMA *: Chromosome microarray analysis
*CS *: Chromosome karyotyping
*NGS *: Next-generation sequencing
*SSP *: Semiconductor sequencing platform
